# Trichain cationic lipids: the potential of their lipoplexes for gene delivery†
†Electronic supplementary information (ESI) available. See DOI: 10.1039/c8bm00965a


**DOI:** 10.1039/c8bm00965a

**Published:** 2018-10-16

**Authors:** Laila Kudsiova, Atefeh Mohammadi, M. Firouz Mohd Mustapa, Frederick Campbell, Katharina Welser, Danielle Vlaho, Harriet Story, David J. Barlow, Alethea B. Tabor, Helen C. Hailes, M. Jayne Lawrence

**Affiliations:** a Institute of Pharmaceutical Sciences. Faculty of Life Sciences and Medicine , King's College London , Franklin-Wilkins Building , Stamford Street , London SE1 9NH , UK; b Department of Chemistry , University College London , Christopher Ingold Laboratories , 20 Gordon Street , London WC1H 0AJ , UK . Email: h.c.hailes@ucl.ac.uk; c Division of Pharmacy and Optometry , School of Health Sciences , Faculty of Biology , Medicine and Health , Stopford Building , University of Manchester , Oxford Road , Manchester , M13 9PT , UK . Email: jayne.lawrence@manchester.ac.uk

## Abstract

Lipoplexes (LDs) have been prepared from DNA and positively charged vesicles composed of the helper lipid, dioleoyl l-α-phosphatidylethanolamine (DOPE) and either a dichain (DC) oxyethylated cationic lipid or their corresponding novel trichain (TC) counterpart. This is the first study using the TC lipids for the preparation of LDs and their application. Here the results of biophysical experiments characterising the LDs have been correlated with the *in vitro* transfection activity of the complexes. Photon correlation spectroscopy, zeta potential measurements and transmission electron microscopy studies indicated that, regardless of the presence of a third chain, there were little differences between the size and charge of the TC and DC containing LDs. Small angle neutron scattering studies established however that there was a significant conformational re-arrangement of the lipid bilayer when in the form of a LD complex as opposed to the parent vesicles. This re-arrangement was particularly noticeable in LDs containing TC lipids possessing a third chain of C_12_ or a longer chain. These results suggested that the presence of a third hydrophobic chain had a significant effect on lipid packing in the presence of DNA. Picogreen fluorescence and gel electrophoresis studies showed that the TC lipids containing a third acyl chain of at least C_12_ were most effective at complexing DNA while the TC lipids containing an octanoyl chain and the DC lipids were least effective. The transfection efficacies of the TC lipids in the form of LDs were found to be higher than for the DC analogues, particularly when the third acyl chain was an octanoyl or oleoyl moeity. Little or no increase in transfection efficiency was observed when the third chain was a methyl, acetyl or dodecanoyl group. The large enhancement in transfection performance of the TC lipids can be attributed to their ability to complex their DNA payload. These studies indicate that presence of a medium or long third acyl chain was especially beneficial for transfection.

## Abstract 2

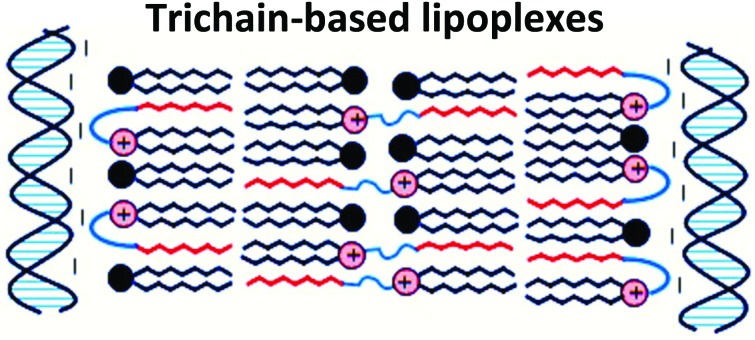
Lipoplexes (LDs) have been prepared from DNA, DOPE and either a dichain oxyethylated cationic lipid or their novel trichain (TC) counterpart.

## Introduction

Lipoplexes (LDs) formed from the complexation of positively charged vesicles formed by synthetic cationic lipids (or cytofectins), alone and in the presence of a neutral co- (or helper) lipid, and negatively charged DNA have been amongst the most widely studied of non-viral vectors.^1^ Interest in LDs stems from their low immunogenicity, their ease of preparation and handling and, importantly their ability to be targeted to specific cell types in the body.^2^,^3^ Unfortunately the commercial exploitation of LDs has been impeded largely because of their low transfection ability, especially when compared to viral vectors.^3^ In an attempt to overcome this limitation, research groups, including our own, have synthesized novel cationic lipids with a view to preparing LDs with an improved transfection ability.^4^–^7^ These studies have demonstrated that it is possible to get improved transfection activity through the careful selection of the cationic lipid used and optimisation of the LD formulation, in particular the charge ratio, type and ratio of neutral co-lipid added and whether the cationic lipid is PEGylated. However, further improvement in transfection ability is required if LDs are to prove to be a realistic, viable alternative to viral vectors.

The present work examines the biophysical and transfection properties of LDs formed using one of two groups of hydroxyl terminated short *n*-ethylene glycol (oxyethylated dichain (DC) derivatives) lipids or their corresponding trichain (TC) counterparts (Table 1). The synthesis of the Group 1 and 2 lipids, together with the biophysical and *in vitro* transfection ability of lipopolyplexes (LPDs) formulated from these lipids has been detailed in Mohammadi *et al.*^8^ Of importance to the present study, the TC lipid-containing LPDs demonstrated an enhancement in transfection performance.^8^ It was of interest whether a similar enhancement in transfection efficiency could be achieved with LDs prepared using the same novel lipids and these were examined in the present study.

**Table 1:** Summary of the dichain and trichain lipids prepared (Group 1 lipids) and dichain lipids capped with different ester chain lengths (Group 2 lipids).^8^

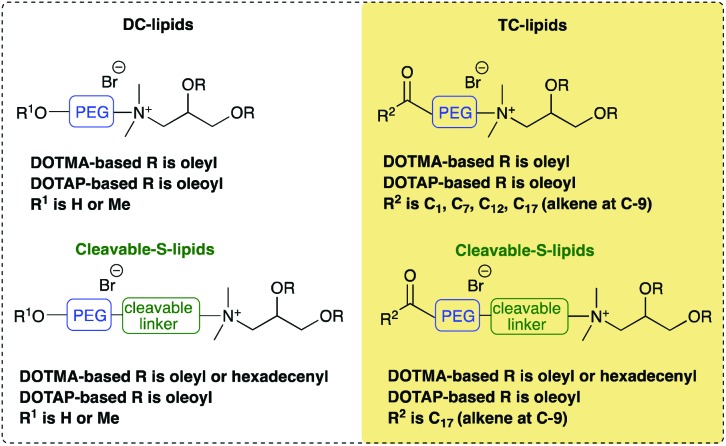			
DOTMA-based ether series	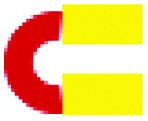	DOTAP-based ester series	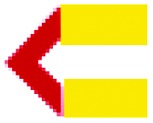
**Group 1 lipids**			
DODEG4	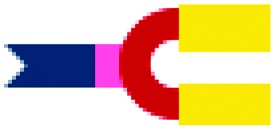	DOesDEG4	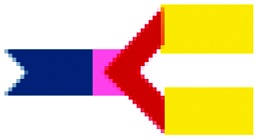
TC-DODEG4	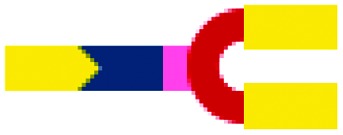	TC-DOesDEG4	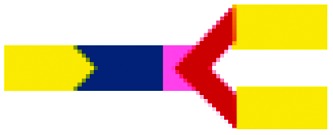
		Me-DOesDEG3	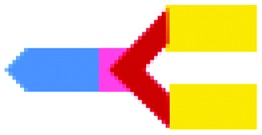
DOSEG3	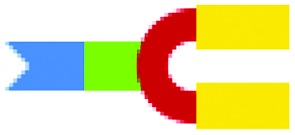	DOesSEG3	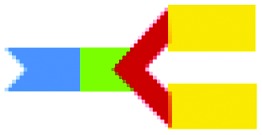
TC-DOSEG3	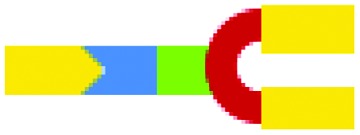	TC-DOesSEG3	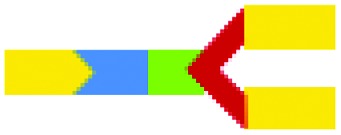
		Me-DOesSEG3	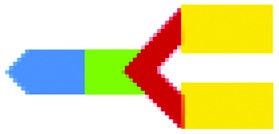
TC-DHSEG3	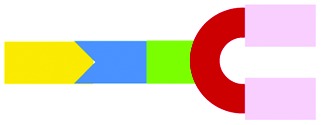		

**Group 2 lipids**			
AC-DODEG4	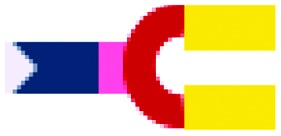	AC-DOesDEG4	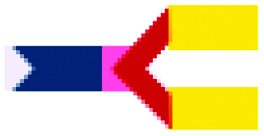
OC-DODEG4	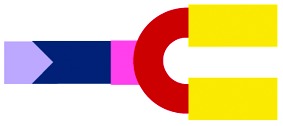		
DO-DODEG4	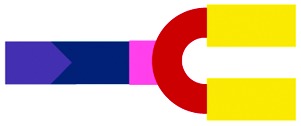	DO-DOesDEG4	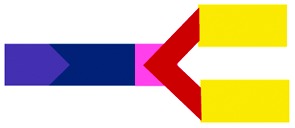
TC-DODEG4	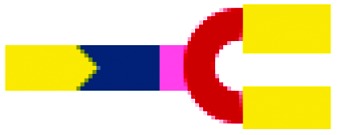	TC-DOesDEG4	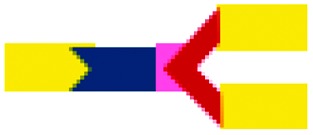

In Group 1 lipids, the cationic moieties of the DC variants were terminated with a short *n*-ethylene glycol (*n*-EG) chain, which itself contained either a free hydroxyl group or was capped with a methoxy group. In the corresponding Group 1 TC lipids, the terminal hydroxyl group was terminated by an unsaturated C_18_ fatty acid. In Group 1 we were therefore able to explore the effect of the presence of an extra oleoyl chain on *in vitro* transfection. The only exception to this was TC-DHSEG3 where two C16:1 chains replaced the C18:1 lipidic chains. In the Group 2 TC cationic lipids, the effect of varying the length of the third fatty acid from acetyl through octanoyl and dodecanoyl to oleoyl was explored, while keeping the other two chains as C18:1. The parent structure of the DC ether and ester lipids were the DOTMA and DOTAP motifs, respectively. All lipids contained only a single cationic charge and were prepared as the bromide salts.

The molecular structure of the novel cationic lipids is described by a short letter code (Table 1). When the prefix **TC**- is used, the lipid is a TC lipid, whereas when no prefix is present, the lipid is a dichain (DC) lipid. The nature of the acyl chains on the basic DC lipid is indicated by the letters **DO** when two oleyl chains are present and **DH** when hexadecenyl chains are present. The nature of the third chain when present is described by **AC**, **OC** or **DO**, for acetyl, octanoyl or dodecanoyl groups, respectively. The molecular structure of the cationic lipids was varied by altering the linking group to the glycerol backbone, either an ester (described using **es**) or an ether moiety. Similarly, the nature of the group attaching the cationic head group to the *n*-ethylene glycol component was varied, being either a short ester spacer (**S**) or direct attachment of the glycol unit to the dimethylammonium cation (**D**). The short ethylene glycol chain present in all the lipids was indicated by either **EG3** or **EG4** depending upon the length of the chain.

The present paper reports the transfection efficiency and the biophysical properties of the LDs formed by a series of novel TC lipids and their corresponding DC lipids and relates them to lipid molecular structure. A detailed and comprehensive understanding of which molecular features are important for transfection is a prerequisite for the development of pharmaceutically sound and efficient LDs. The interaction of vesicles containing these novel cationic lipids in combination with a 1 : 1 molar ratio of dioleoyl l-α-phosphatidylethanolamine (DOPE) with DNA (either plasmid or calf thymus) was investigated using several biophysical techniques and the biophysical properties of the resulting LDs then correlated with their *in vitro* lipofection activity. The properties of LDs formed using the Group 1 and Group 2 TC lipids were of particular interest in the present study because of the major enhancement in cellular transfection performance observed with LPDs formulated containing these novel TC lipids. Significantly, to our knowledge in this study it is the first time that TC lipids have been used for the preparation of LDs.

## Results

### Biophysical characterisation of the vesicles

#### Vesicles: photon correlation spectroscopy and zeta potential measurements

The formation and characterisation of vesicles prepared by DOTMA and DOTAP and their *n*-EG DC and TC derivatives (*i.e.* Group 1 and 2 lipids) when in combination with a 1 : 1 molar ratio of DOPE using photon correlation spectroscopy, zeta potential, electron microscopy and small angle neutron scattering have been previously reported by Mohammadi *et al.*^8^ In this earlier study it was reported that it was not possible to form vesicles using DO-DODEG4 or its ester equivalent DO-DOesDEG4 when in combination with a 1 : 1 molar ratio of DOPE. It did, however, prove possible to prepare stable vesicles from both of these lipids when used in combination with a 1 : 1 molar ratio of DOPC. This interesting observation is in agreement with the known tendency of DOPE to form non-bilayer structures.^9^ Vesicles prepared from DO-DODEG4 and DO-DOesDEG4 were therefore formulated with 50 mol% DOPC in place of DOPE. Furthermore, it should be noted that earlier SANS, TEM and size measurement experiments on similar complexes showed no difference in the internal structure or the size/shape of lipoplexes, when plasmid DNA or calf thymus DNA (ctDNA) were used.^6^ ctDNA was therefore used in experiments requiring large amounts of DNA, where the use of plasmid was not practical.

**Fig. 1:**
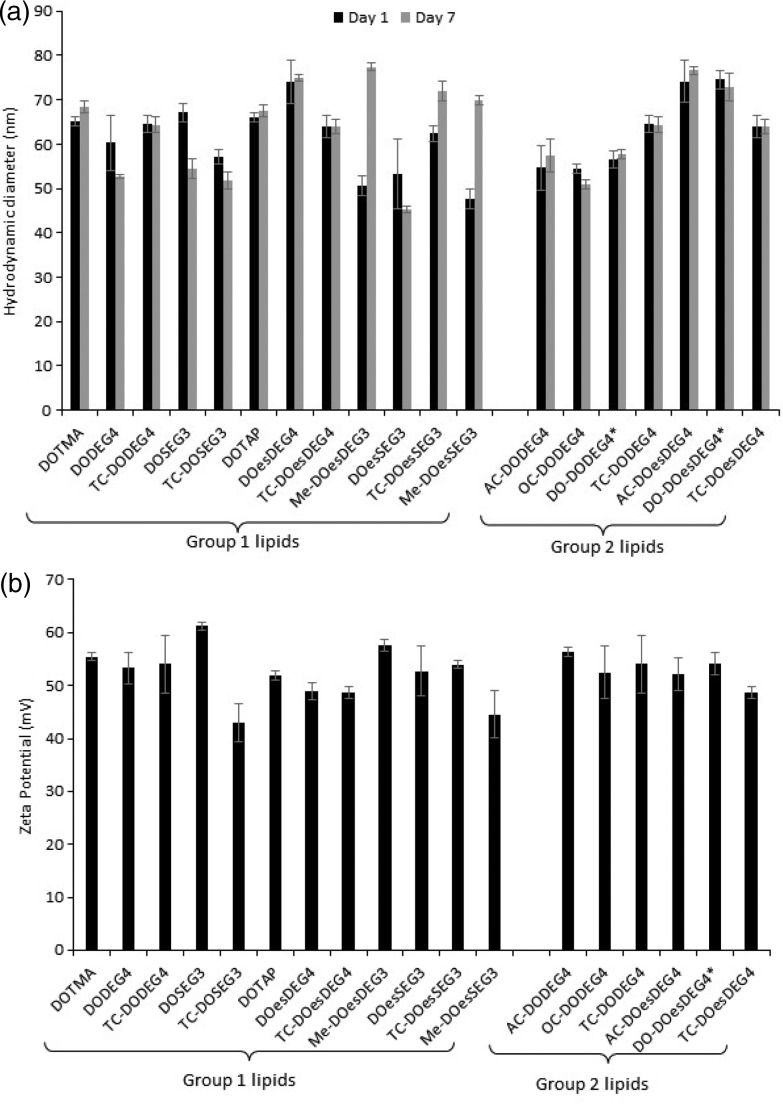
(a) The apparent hydrodynamic size (nm) of vesicles on the day of their preparation (day 1) (black bars) and 7 days after their preparation (day 7) (grey bars) and (b) the zeta potential (mV) of the same vesicles measured on the day of their preparation. All vesicles were prepared from cationic lipids in combination with DOPE or (*) DOPC at a cationic lipid concentration of 0.01 mg mL^–1^. Measurements were performed at 298 ± 0.1 K. in triplicate and shown as the mean value ± standard deviation.

Fig. 1(a) illustrates the apparent hydrodynamic size of the LD vesicles formed using the Group 1 and 2 lipids. As expected from the nature of the preparation, the sizes, although slightly different from those previously reported, are comparable.^8^ As can be seen, the apparent hydrodynamic sizes of the parent vesicles, assuming the presence of spherical particles, were in the range 45–80 nm. The vesicles were stable with respect to size for a period of at least a week (Fig. 1(a)) and generally up to one month (Fig. 1, ESI†). It should be noted that vesicles were prepared from the DC and TC-lipids on a number of occasions and while the vesicle sizes fell within the range quoted above, there was no reproducible trend in the relationship between lipid structure and vesicle size. However, the TC-lipids tended to form vesicles at the upper end of the size range. The relatively small size of the vesicles (*i.e.* all <100 nm), indicated that they were likely to be predominately unilamellar in nature, particularly those containing DC-lipids which were at the lower end of the vesicle size range. It is not possible however to exclude the presence of minor populations of multilamellar within the preparations.

**Fig. 2:**
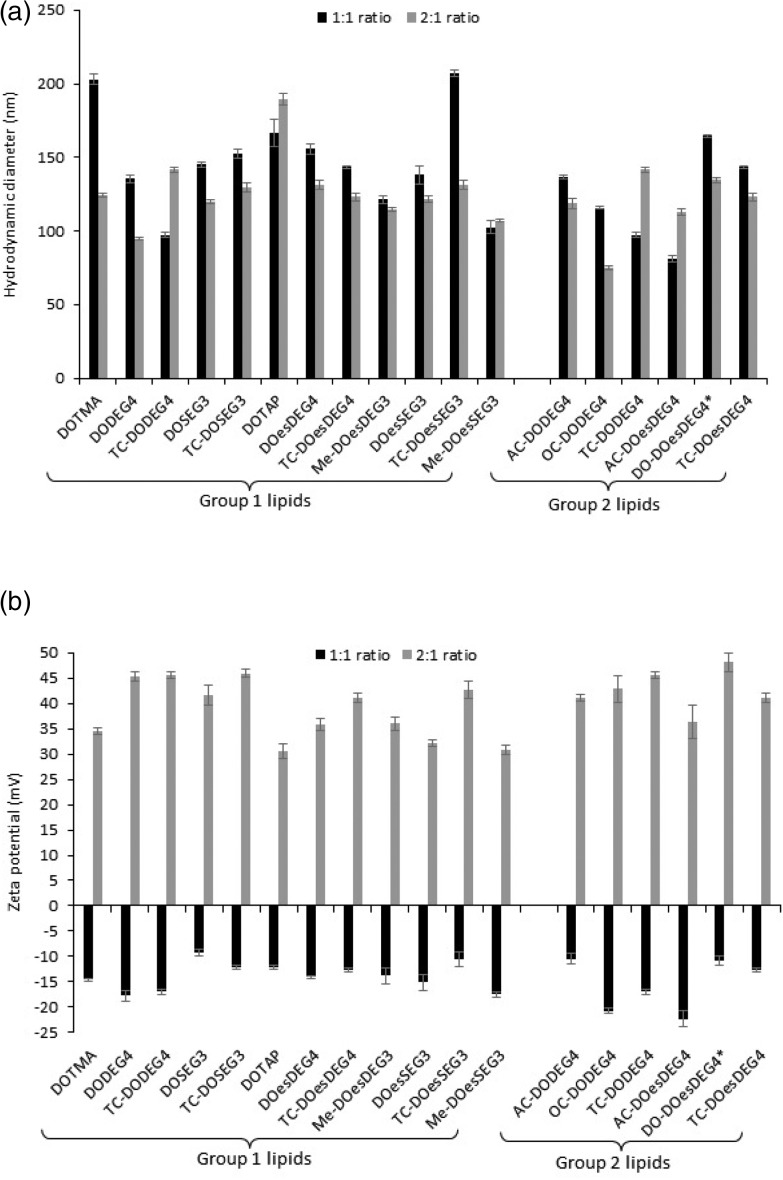
(a) The apparent hydrodynamic size (nm) and (b) zeta potential (mV) of LDs prepared from vesicles of the cationic lipids in combination with either DOPE or (*) DOPC and ctDNA at L : D charge ratios of either 2 : 1 (grey bars) or 1 : 1 (black bars), respectively, and a ctDNA concentration of 1 μg mL^–1^. All measurements were performed in triplicate at 298 ± 0.1 K on the day of preparation and are shown as the mean value ± standard deviation.

A limited study was performed to look at the effect of pH (either 7.4 or 5) on the size of the vesicles formed by a selection of DC and TC lipids, namely DODEG4, TC-DODEG4, DOSEG3 and TC-DOSEG3 in combination with a 1 : 1 molar ratio of DOPE. As expected (Fig. 2, ESI†) there was no relationship between vesicle size and pH of the aqueous solvent used for its preparation. All vesicles possessed a zeta potential of at least ∼40 mV (Fig. 1b) supporting the good stability observed with the vesicles by photon correlation spectroscopy. Indeed, the vesicles were stable with respect to zeta potential for a period of up to one month (Fig. 1, ESI†). Again, there was no clear trend between lipid type and vesicle zeta potential, with the absolute value determined varying slightly each time the vesicles were prepared.

#### Vesicles: small angle neutron scattering

**Fig. 3:**
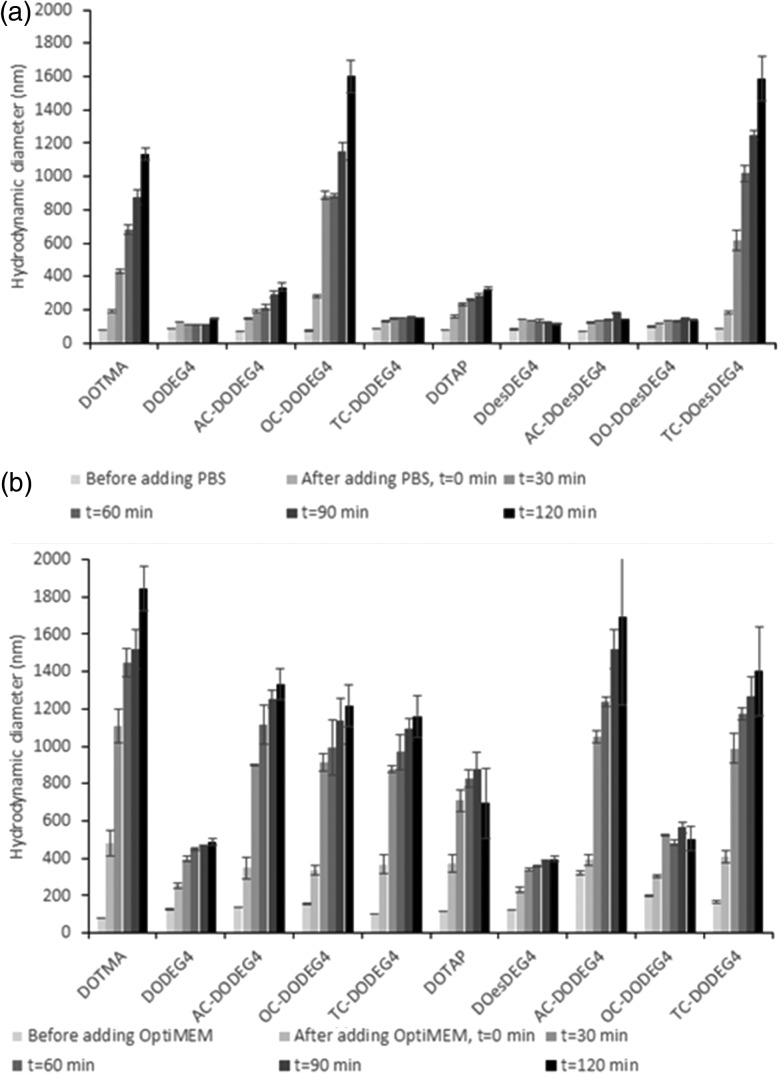
The variation in apparent hydrodynamic size (nm) of LDs, prepared from vesicles of the various lipids in combination with DOPE or (*) DOPC using ctDNA at L : D charge ratios of 2 : 1 in the presence of either (a) PBS or (b) Opti-MEM as a function of incubation time. Measurements were performed at a DNA concentration of 1 μg mL^–1^ for the LDs. All measurements were performed in triplicate at 298 ± 0.1 K and are shown as the mean value ± standard deviation.

Table 2 gives the structural parameters used to obtain the best fit to the small angle neutron scattering (SANS) data for freshly prepared vesicles containing 50 mol% DOPE and a novel cationic lipid (Group 1 or Group 2) at cationic lipid concentrations of 1 mg mL^–1^. Despite their small apparent hydrodynamic size, the vesicles were well modelled as single, flat sheets (or bilayers) with or without the presence of (multilamellar) stacks. The use of a flat sheet model to analyse the SANS data here is in agreement with earlier SANS studies on vesicles of comparable hydrodynamic size.^10^,^11^ An example of the SANS profile recorded for the vesicles prepared by the dichain cationic lipid (Me-DOesDEG3) and DOPE along with the best fit to the data obtained using a flat sheet model is shown in Fig. 3, ESI.† Significantly, the SANS studies on the vesicles indicated that, regardless of the chemical structure of the novel lipid, the thicknesses of the various vesicle bilayers was in the range 3.8–4.3 nm. When the presence of bilayer stacks were required to model the data, the proportion of multilamellar vesicles was very low (supporting the presence of a predominately unilamellar vesicle population), while the measured repeat distance was 5.7–5.8 nm with the exception of a couple of the Group 1 TC lipids (TC-DOesDEG4 and TC-DOesSEG3) where a slightly larger repeat was observed (Table 2). As noted previously^8^ there was no correlation of the thickness of the bilayer with lipid structure – namely bond type (ester or ether), length of the *n*-EG linker and the absence/presence of a third chain irrespective of its length. As a consequence of the similarity of the parameters obtained for the vesicles, it was concluded that TC lipid-containing aggregates prepared under the conditions used were vesicular. Reassuringly, the parameters obtained from fitting the SANS results of the vesicles in this study are similar to those reported for the LPD systems.^8^

**Table 2:** Structural parameters obtained through model fitting of the small angle neutron scattering data recorded at 298 ± 0.1 K for freshly prepared cationic vesicles containing the novel lipids together with a 1 : 1 molar ratio of DOPE or (*) DOPC (V) at a cationic lipid concentration of 1 mg mL^–1^, and corresponding lipoplexes (LD) prepared from the vesicles and ctDNA at a L : D charge ratio of 2 : 1 at a cationic lipid concentration of 0.1 mg mL^–1^

Cationic lipid	*L* (nm)	*D* (nm)
DOTMA	_V_4.2 ± 0.1	_V_6.2 ± 0.1
	_LD_4.2 ± 0.2	_LD_6.5 ± 0.1
DODEG4	_V_4.1 ± 0.1	ND
	_LD_5.4 ± 0.2	_LD_6.4± 0.2
TC-DODEG4	_V_4.2 ± 0.1^*a*^	_V_5.8 ± 0.1^*a*^
	_LD_12.7 ± 0.3	_LD_14.3 ± 0.1
DOSEG3	_V_4.2 ± 0.1	ND
	_LD_4.9 ± 0.2	_LD_5.8 ± 0.1
TC-DOSEG3	_V_4.1 ± 0.1	_V_5.7 ± 0.1
	_LD_10.3 ± 0.2	_LD_13.5 ± 0.1
TC-DHSEG3	_V_4.2 ± 0.1	_V_5.8 ± 0.1
	_LD_11.3 ± 0.1	_LD_14.0 ± 0.1
DOTAP	_V_4.0 ± 0.1	_V_6.1 ± 0.1
	_LD_4.1 ± 0.2	_LD_6.0 ± 0.1
DOesDEG4	_V_3.9 ± 0.1	_V_5.7 ± 0.1
	_LD_5.4 ± 0.2	_LD_6.4 ± 0.1
TC-DOesDEG4	_V_4.1 ± 0.1	_V_6.5 ± 0.3
	_LD_10.7 ± 0.4	_LD_14.3 ± 0.1
Me-DOesDEG3	_V_4.0 ± 0.1^*b*^	_V_5.7 ± 0.1^*b*^
	_LD_5.4 ± 0.5	_LD_6.4 ± 0.1
DOesSEG3	_V_3.8 ± 0.1	_V_5.7 ± 0.1
	_LD_5.3 ± 0.3	_LD_6.5 ± 0.1
TC-DOesSEG3	_V_4.3 ± 0.1	_V_6.0 ± 0.1
	_LD_10.3 ± 0.4	_LD_13.5 ± 0.1
Me-DOesSEG3	_V_4.0 ± 0.1^*b*^	ND
	_LD_5.4 ± 0.3	_LD_6.4 ± 0.1
AC-DODEG4	_V_3.9 ± 0.1	ND
	_LD_8.2 ± 0.3	_LD_12.0 ± 0.3
OC-DODEG4	_V_3.8 ± 0.1	_V_5.9 ± 0.1
	_LD_9.4 ± 0.4	_LD_12.9 ± 0.2
DO-DODEG4*	_V_3.9 ± 0.1	ND

^*a*^ *M* (the number of stacks in a multilayer structure) = 5.

^*b*^ *M* = 4, in all other cases *M* = 20.

### Biophysical characterization of the lipoplexes

#### Lipoplexes: photon correlation spectroscopy and zeta potential measurements

As can be seen from Fig. 2a, the apparent hydrodynamic size of the LD complexes varied from ∼75 nm (LDs containing OC-DODEG4 at a 2 : 1 charge ratio) to just over 200 nm (LDs containing TC-DOesSEG3 at a 1 : 1 charge ratio), with most lipoplexes being in the approximate size range of 125–175 nm. As was observed with the LPDs there was no correlation between the size of the final LD and the structure of the cationic lipid it contained. Furthermore, while all LDs prepared at a L : D charge ratio of 2 : 1 exhibited a highly positive zeta potential, all the lipoplexes (regardless of cationic lipid structure) when prepared at a 1 : 1 charge ratio exhibited a negative zeta potential of between –9 and –22 mV (Fig. 2b), suggesting that not all the negative charges of the DNA had been neutralized by the cationic lipid.

**Fig. 4:**
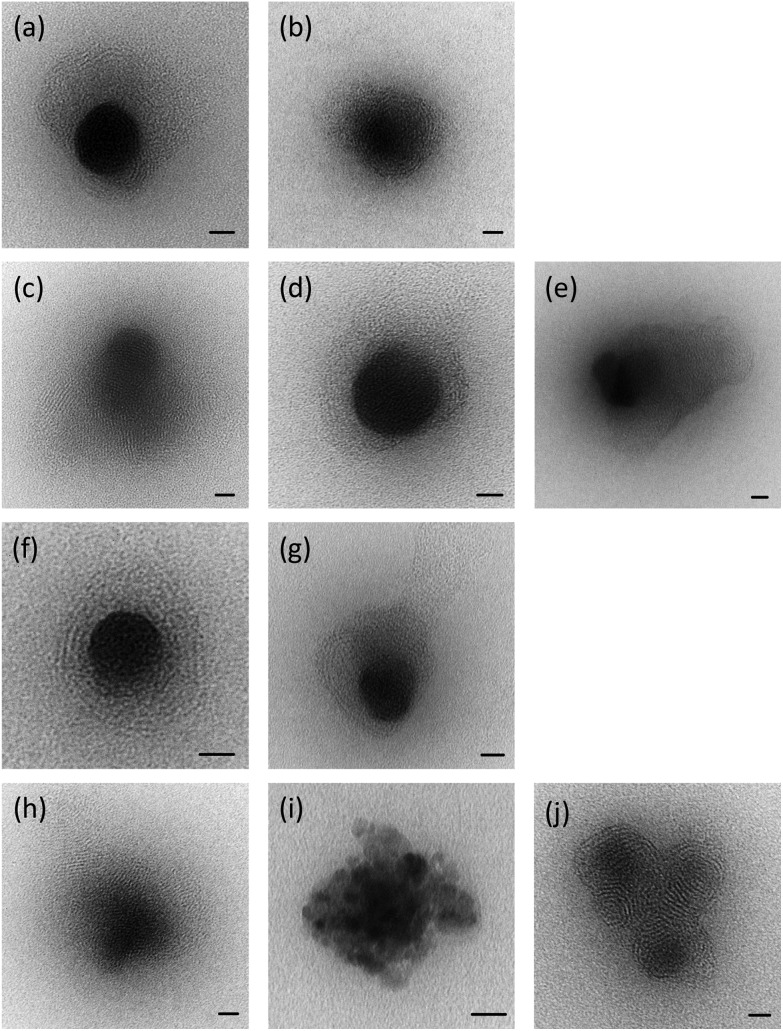
Transmission electron microscopy of LD complexes (lipoplexes prepared using ctDNA at a cationic lipid : DNA charge ratio of 2 : 1 made with (a) DODEG4; (b) DOesDEG4; (c) TC-DODEG4; (d) TC-DOesDEG4; (e) Me-DOesDEG3; (f) DOSEG3; (g) DOesSEG3; (h) TC-DOSEG3; (i) TC-DOesSEG3; (j) Me-DOesSEG3. Size bar = 20 nm.

Lipoplex stability towards either PBS or Opti-MEM media was studied by establishing the variation in apparent hydrodynamic size (nm) over time of LDs prepared at a 2 : 1 L : D charge ratios and containing a selection of Group 1 and Group 2 lipids (Fig. 3). Fig. 4, ESI† shows the apparent hydrodynamic size of a range of LDs at a 1 : 1 and 2 : 1 charge ratio in the presence of Opti-MEM and FBS-containing media. Notably, the LDs were more stable in PBS than in Opti-MEM, with only LDs containing either of the TC-lipids, OC-DODEG4 and TC-DOesDEG4 or DOTMA exhibiting a high degree of instability in PBS, increasing to more than a 1 μm in size over the 2 h incubation period (Fig. 3). All other LDs tended to be much more stable under the conditions of the test. In contrast, most LD complexes incubated in Opti-MEM, and to a much lower extent FBS-containing media (Fig. 4 ESI†), increased their size over the same period, although it was noticeable that the LDs containing the DC lipids, DODEG4, DOesDEG4 and DO-DOesDEG4, grew in size the least, suggesting that they produced the most stable LD complexes, probably due to the steric stabilising effect of the exposed *n*-EG chain.

#### Lipoplexes: small angle neutron scattering

The parameters used to obtain the best fit to the SANS data for all LDs prepared at a L : D charge ratio of 2 : 1 using ctDNA prepared using Group 1 and 2 cationic lipids (measured at a cationic lipid concentration of 0.1 mg mL^–1^) are shown in Table 2. It was not possible to model any of the LDs as comprising just single sheets (*i.e.* bilayers). Instead it was necessary to invoke the stack model to obtain a good fit to the SANS data, suggesting that the lipoplexes possessed repeating bilayers of lipid between which the DNA was intercalated.^12^ Fig. 3, ESI† gives an example of the SANS profile obtained for an LD prepared using vesicles of the dichain cationic lipid (Me-DOesDEG3) and DOPE and ctDNA along with the best fit to the data using the mixed sheet and stack model. Significantly, the thickness of the bilayer was much greater when the cationic lipids were part of a lipoplex, as opposed to when the lipids were in the form of vesicles. With the exception that LDs containing either DOTMA or DOTAP, where bilayer thickness did not change in the presence of DNA, the thickness of all the bilayers containing the DC lipids, including the methoxy capped DC lipids increased by up to 1.4 nm in the presence of DNA, suggesting a significant re-arrangement of the lipids was necessary to accommodate the interaction of the cationic group with the negative charges on DNA.

All TC lipids in Group 1 and Group 2 exhibited a much greater increase in bilayer thickness, with increases of ∼4–5 nm being observed for the AC- and OC-lipids and in the range of 6–9 nm for the TC-lipids, again suggesting a significant structural re-arrangement of the packing of lipids when in a lipoplex. However, it is not clear what the precise conformational arrangement of the lipids within the expanded ‘bilayer’ are, or indeed if the layer is still forming of a traditional bilayer. In this respect the repeat distances generally yielded a water layer in the approximate range of 1.6–1.9 nm for the DC lipids and between 1.6–3.2 nm for the TC lipids. It is within this water layer that the DNA would be expected to reside.

A re-arrangement of the same *n*-EG cationic lipids has been previously noted when the lipids were in lipopolyplexes (a complex of lipid, targeting peptide and DNA (LPD)).^8^ In this instance, however, the change in bilayer thickness was less, most probably because of the different structures adopted by the LD and LPD complexes. In contrast, previous SANS studies on vesicles and their corresponding LD and LPDs prepared using non-*n*-EG cationic lipids, including DOTMA and DOTAP (as also shown in our LD results in Table 1) did not exhibit any discernable difference in bilayer thickness between the vesicles and LDs/LPDs,^4^–^8^ illustrating the effect of the presence of an *n*-EG group on packing in the gene delivery vehicles.

#### Lipoplexes: transmission electron microscopy

The LD complexes prepared using a selection of TC and DC (Group 1) lipids at a L : D charge ratio of 2 : 1 using ctDNA were visualised using negative staining transmission electron microscopy (TEM). A selection of the micrographs is shown in Fig. 4. It was clear that, in the presence of DNA, the lipid vesicles had undergone a re-arrangement to form either larger individual particles or large clusters thereof, each of which possessed an electron-dense core, containing and surrounded by a regular repeating “lamellar-like” structure, believed to be composed of lipid layers, between which electrostatically-bound DNA is sandwiched. The presence of multilamellar structures for the LD complexes is in agreement with the results of the SANS study and the larger size of the lipoplexes compared to their parent vesicles measured by dynamic light scattering. Although the LDs were very polydisperse in size, there was no obvious difference between the nature or average size of structures containing the various cationic lipids. It is of note that no (uncomplexed) vesicles were detected by TEM in the LD complexes, suggesting that all of the lipid formed complexes.

### Lipoplexes: picogreen fluorescence studies

**Fig. 5:**
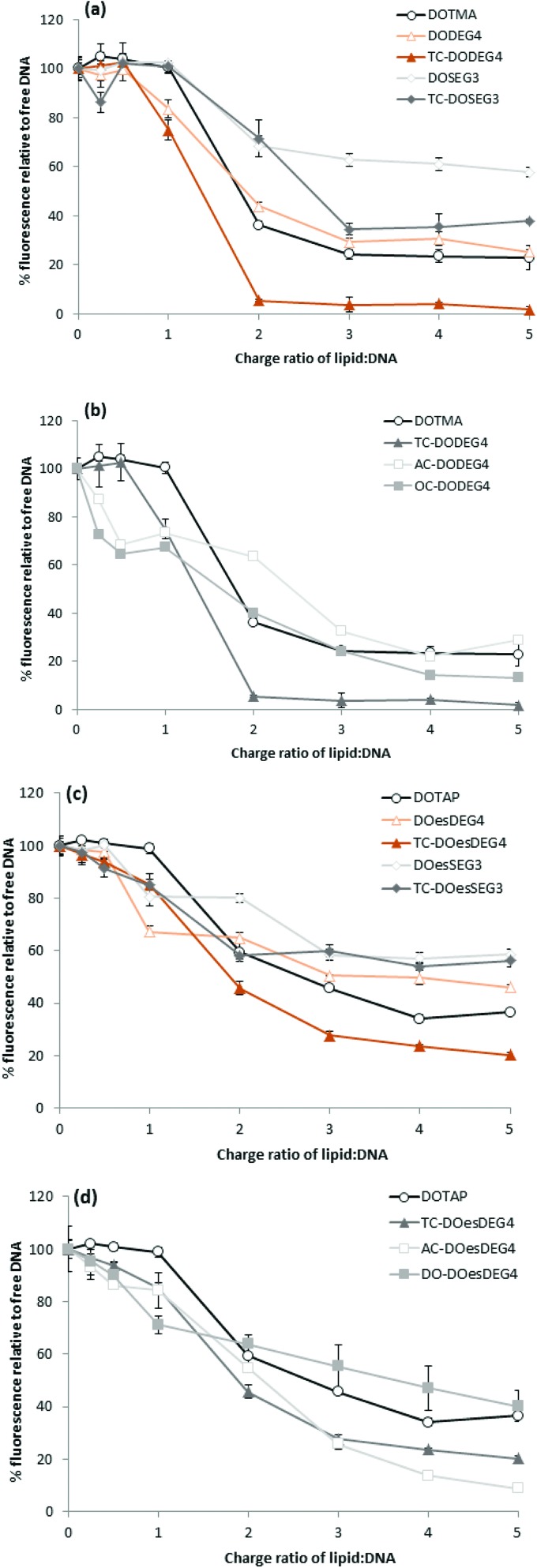
Picogreen fluorescence binding assay showing pDNA packaging with LD complexes containing (a) Group 1 DOTMA lipids; (b) Group 2 DOTMA lipids; (c) Group 1 DOTAP lipids and (d) Group 2 DOTAP lipids and prepared with varying L : D charge ratios of 0.25 : 1–5 : 1. Relative fluorescence units (RFU) of the LD complexes were calculated as percentage fluorescence from free DNA.

Picogreen fluorescence studies were performed on both Group 1 and 2 lipids in an attempt to study the efficacy of the packaging of pDNA within the LD complexes at L : D charge ratios of between 0.25 : 1 and 5 : 1 (Fig. 5). As anticipated the interaction of the cationic lipids with DNA results in a reduction of the fluorescence intensity of picogreen, ultimately reaching a plateau (suggesting that maximum complexation had been reached for that complex). The lower the plateau fluorescence intensity reached, the stronger the DNA condensation.^13^ For the LD complexes prepared using Group 1 lipids, the plateau (or maximum complexation) was reached at a L : D charge ratio of 2 : 1–3 : 1, while for the Group 2 lipids containing either an AC-or OC-third chain, the plateau was achieved at a slightly higher L : D charge ratio of 3 : 1–4 : 1, suggesting that these lipids were not as effective at condensing pDNA. From a comparison of the value of the plateau in fluorescence intensities shown in Fig. 5, it is also obvious that the long oleoyl chain TC-lipids are more effective at complexing DNA than their corresponding DC-lipids, and that lipids in which the *n*-EG group was directly linked to the headgroup (*i.e.* DODEG4, TC-DODEG4, DOesDEG4 and TC-DOesDEG4) were consistently better at condensing DNA compared to their corresponding lipids containing a cleavable linker (DOSEG3, TC-DOSEG3, DOesSEG3 and TC-DOesSEG3). It could be envisaged that the short *n*-EG chain (and potentially also the cleavable ester linker) may generate a thin hydrophilic barrier, resulting in a slightly greater distance between the cationic charge and the negatively charged DNA, resulting in a weaker interaction between the two oppositely charged moieties. In contrast, when a third chain is introduced on the terminal hydroxyl of an *n*-EG chain, the hydrophobic chain could be embedded in the lipid bilayer due to a hairpin bend in the oxyethylene chain, bringing the cationic charge closer to the anionic DNA and thereby facilitating their interaction.

### Lipoplexes: gel electrophoresis

**Fig. 6:**
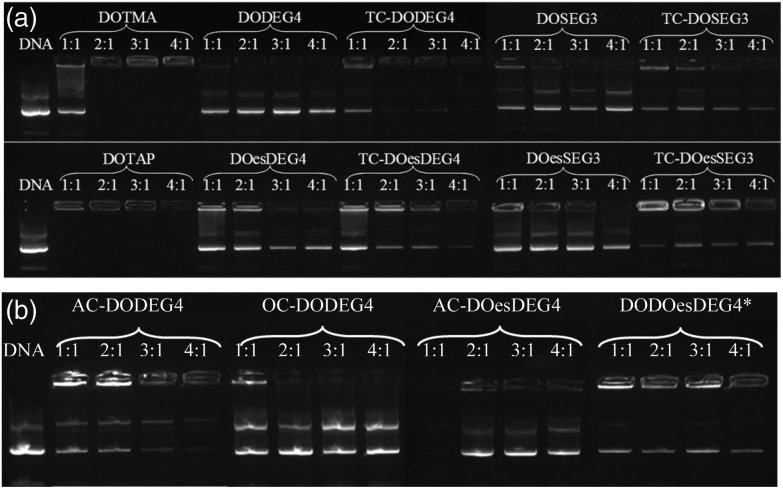
Gel condensation LDs prepared from (a) Group 1 and (b) Group 2 lipids with a 1 : 1 ratio of DOPE or *DOPC prepared at 1 : 1 to 4 : 1 charge ratio with DNA.

Gel electrophoresis was performed on the LD complexes prepared using the DC and TC lipids from Groups 1 and 2 at L : D charge ratios of 1 : 1 to 4 : 1 to determine the extent of complexation of pDNA (Fig. 6). In agreement with the picogreen fluorescence studies, the TC lipids of Group 1, were more effective at complexing pDNA than their DC equivalents, (indeed, even at the higher L : D charge ratio of 4 : 1, none of the DC lipids appeared to completely complex the DNA). Furthermore, lipids with the *n*-EG chain directly linked to the headgroup were also more effective at condensing DNA compared to their corresponding lipids containing the cleavable linker for reasons discussed above. DOTMA and DOTAP, however, still showed the best DNA complexation using this technique. Amongst the Group 2 lipids, AC-DODEG4 and DO-DOesDEG4 were the most effective at complexing pDNA, with AC-DODEG4 being almost as effective as the TC lipids of Group 1.

#### Lipoplexes: *in vitro* transfection

**Fig. 7:**
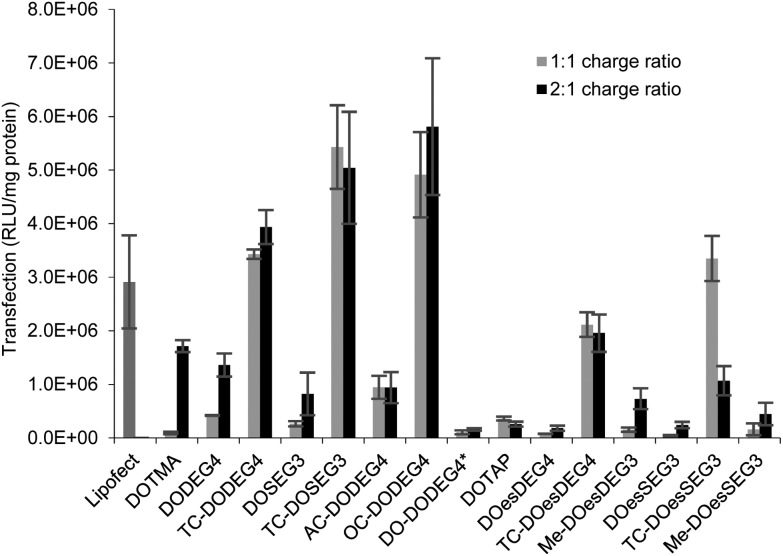
*In vitro* transfection efficiency (expressed as RLU mg^–1^ protein) of LD complexes prepared in Opti-MEM at 1 : 1 (grey bars) and 2 : 1 (black bars) lipid : gWiz plasmid DNA charge ratio in B104 cells. Cationic lipid used to prepare the LDs shown on the abscissa. All the lipids were formulated at a 1 : 1 ratio with DOPE or (*)DOPC. Data are the mean of three measurements ± standard deviation.

The majority of transfections were performed in rat neuroblastoma B104 cells. This cell line was selected as it is intended to use the lipoplexes produced in the present study for the treatment of neurodegenerative diseases. Specifically, the lipoplexes will be delivered to the brains of rats *via* convection enhanced delivery.^15^ LDs prepared from mixing vesicles containing either a TC lipid, or the corresponding DC analogue, in the presence of a 1 : 1 molar ratio of DOPE with gWiz plasmid DNA in either a 1 : 1 or a 2 : 1 L : D charge ratio (Fig. 7) were tested. The LDs were prepared in Opti-MEM thereby allowing a comparison with the results of other groups.^14^,^15^ Note, that although we include data from only one set of transfection experiments here, these experiments were repeated on at least three occasions and while the absolute values obtained for transfection efficiency varied, the trends observed between data sets were consistent. A similar trend of transfection behaviour to that obtained in B104 cells was seen in mouse neuroblastoma N2A cells. Although higher absolute levels of transfection were achieved (Fig. 5 ESI†), higher cytotoxicity (as assessed by the protein content after exposure of the cells to the lipoplexes) was also seen in this cell line as shown in Fig. 6 ESI.†

Surprisingly, there was little difference observed between the transfections obtained with LDs prepared at the two charge ratios. Note too that regardless of the number of hydrocarbon chains present, LD complexes prepared using the ether lipids, *i.e.* the ‘DOTMA’ series, exhibited higher transfections than those containing ester lipids, *i.e.* the ‘DOTAP’ series. Most significantly in the present study, LD complexes prepared using TC-lipids composed of three C_18_ chains (*i.e.* TC-DODEG4, TC-DODEG3, TC-DOesDEG4, TC-DOesEG3) exhibited a reproducibly higher level of transfection than their corresponding DC-chain lipids (DODEG4, DODEG3, DOesDEG4, DOesEG3). In addition, they were superior transfection agents compared to Lipofectamine and LD complexes containing either DOTMA or DOTAP. Capping the terminal hydroxyl of a DC-lipid with a methyl group had the effect of slightly increasing the transfection ability (Me-DOesDEG4, Me-DOesEG3) but not to the extent achieved using long oleoyl chain TC lipids. Interestingly, when considering the Group 2 ‘DOTMA’ lipids, addition of an acetyl group (AC-DODEG4) appeared beneficial to the transfection, but not as beneficial as the addition of an octanoyl chain (OC-DODEG4). Surprisingly, lengthening of the chain attached to the terminal hydroxyl group to dodecanoyl (DO-DODEG4) was observed to virtually abolish any transfection, although this may have been predominantly due to the need to replace DOPE with DOPC when preparing the parent vesicles.

The cytotoxicity exhibited by the B104 cells after their incubation with the LDs for 4 h was assessed by determining total protein content after removal of dead cells and media *via* a thorough washing of the cells. The results were compared with those obtained after 4 h incubation with Lipofectamine, where a viability of 72% was achieved. Significantly, when Lipofectamine 2000 was used for transfection, severe reductions in cell viability, were observed and as a consequence, in the present study, Lipofectamine 2000 was not used as a standard. The results of the study determining protein content of the B104 cells after incubation with the LDs are shown in Fig. 7 (ESI†). When incubated with lipoplexes prepared at a 1 : 1 lipid : DNA charge ratio, the cells exhibited a cell viability of greater than 85% when compared to an untreated control. In contrast, incubation of the B104 cells with lipoplexes prepared at a 2 : 1 charge ratio resulted in lower cell viabilities of between 38%–92%. LDs containing TC lipids at the 2 : 1 charge ratio exhibited a reduction in cell viability, which was greatest with the ester (DOTAP) series of lipids, specifically TC-DOesDEG4 and TC-DOesSEG3. In addition, incubation with LDs containing either the Group 2 lipids AC-DODEG4 and OC-DODEG4, or the methoxy capped lipids, Me-DOesDEG4 and Me-DOesEG3, at a 2 : 1 charge ratio also resulted in lower cell viabilities.

## Discussion

**Fig. 8:**
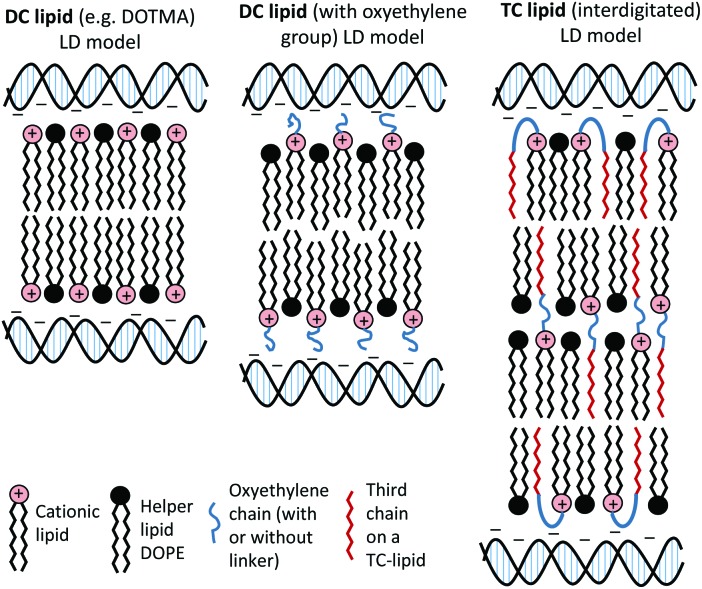
Proposed structural re-arrangement of DC and TC lipids during lipoplex formation.

The present study focused on determining the structural and formulation requirements necessary for obtaining high levels of *in vitro* transfection activity using LDs prepared from a series of novel TC cationic lipids, compared to the corresponding DC lipids. In order to understand what effect the presence of an additional, third hydrophobic chain has on the biophysical properties of the LD complexes, and whether any differences in their biophysical properties could be correlated with differences in transfection, several biophysical experiments have been performed. Although no differences in the size and shape of the LDs formed by the DC and TC lipids could be observed by TEM, small angle neutron scattering studies showed that there was a significant conformational re-arrangement of the lipid bilayer, particularly in the LD complexes prepared using TC lipids possessing a long third alkyl chain. It is thought that this rearrangement was necessary to allow the positive charge on the cationic lipid to interact effectively with the negatively charged DNA.^16^ Studies have been carried out to investigate whether *n*-EG DC cationic lipids undergo such a rearrangement to enable the cationic moiety to interact with the negatively charged phosphate on the DNA.^17^,^18^ Indeed the presence of the *n*-EG group in the DC lipids is thought to introduce a degree of steric repulsion which hinders the electrostatic interaction between the cationic lipid headgroup and DNA (as evidenced by gel electrophoresis and picogreen results conducted using PEG-2000 lipids^18^). This combination of forces could lead to the slight protrusion of the cationic lipids in the DC-lipid : DOPE bilayer, resulting in an overall thicker bilayer (as proposed in the schematic diagram in Fig. 8). In the case of the TC lipid-containing bilayers, which were prepared in combination with DOPE or DOPC during vesicle formation, the third acyl chain may be embedded in the lipid ‘bilayer’ through folding of the *n*-EG chain, especially since no increase in bilayer thickness or multilamellar structures have been observed in vesicles alone, compared to the corresponding DC lipids in SANS studies. In the case of LD complexes, however, the dramatic increase in bilayer thickness for all the TC lipid systems suggests major rearrangements in the lipid bilayers. The thickness of all the TC lipids is around twice that of their corresponding DC lipids. This could suggest that, on average, two bilayers become inter-digitated with the third chain from a lipid in one bilayer being inserted into an adjacent bilayer, and held in place by an *n*-EG bridge (Fig. 8). Indeed several studies^19^,^20^ have proposed that the first step in lipoplex formation involves coating of the vesicles with DNA, followed by interaction with a second DNA-coated vesicle. Afterwards, the two vesicles either fuse or break and roll over each other.^18^ It is therefore possible that during this re-arrangement process, the insertion of the third chain into an adjacent bilayer can occur.

It should be noted that the increase in bilayer thickness in TC-lipid lipoplexes (as suggested by the SANS measurements) was not detected in TEM images (Fig. 4). This is most likely a consequence of the resolution limitations of the imaging instrument, or the presence of the negative stain which might not differentiate between a normal and an interdigitated bilayer. The proposed model in Fig. 8 was therefore based predominantly on the SANS results.

The *in vitro* transfection efficacies of LDs prepared from TC-lipids (especially those possessing a long third acyl chain) were found to be consistently higher than their DC analogues. Whether this difference in transfection is due to the *n*-EG chain protruding out in the case of DC lipids (therefore hindering cellular internalisation and trafficking as most studies have reported when using PEGylated lipids^21^) as opposed to being folded due to the embedding of the third chain, in the case of the TC lipids is unknown at present. However, all the TC-lipid containing lipoplexes also showed significantly better transfection (at least 2–3 fold higher) compared to DOTMA and DOTAP, which do not contain any oxyethylene groups. This suggests that different factors contribute to their superiority. Since the picogreen fluorescence and gel electrophoresis studies showed that the TC lipids were most effective at complexing DNA, it is thought that DNA complexation could play a major role in their enhanced transfection efficiency. In addition, it is possible that the ‘third’ lipid chain may insert into the endosomal membrane, thereby mediating endosomal release and further enhancing transfection.

TC-lipids containing a short third alkyl chain, although not as effective as their long chain counterparts were more effective than the corresponding DC lipids. An interesting exception to these observations was seen with the LDs containing OC-DODEG4 which did not show a strong binding of DNA but did exhibit a good level of transfection. In summary, the TC-lipids reported here show interesting *in vitro* transfection properties and might potentially therefore be developed as a new class of transfection agents for non-viral gene delivery.

## Experimental

Detailed experimental procedures, characterisation data, and ESI Fig. S1–S6 are in the ESI.†

## Conflicts of interest

There are no conflicts of interest to declare.

## Supplementary Material

Supplementary information
