# Supplementary material for: Trichain cationic lipids: the potential of their lipoplexes for gene delivery
Source: Biomater Sci. 2018 Oct 16;7(1):149–58. doi: 10.1039/c8bm00965a (PMC6336150; doi:10.1039/c8bm00965a)
Supplement: Supplementary file 1 [file BM-007-C8BM00965A-s001.pdf]

Electronic Supplementary Material (ESI) for Biomaterials Science.
This journal is © The Royal Society of Chemistry 2018

Trichain Cationic Lipids: The Potential of their Lipoplexes for Gene Delivery

Laila Kudsiova, Atefeh Mohammadi, M. Firouz M. Mustapa, Frederick Campbell, Katharina Welser, Danielle Vlaho, Harriet Storey, David B. Barlow, Alethea B. Tabor, Helen C. Hailes and M. Jayne Lawrence

SUPPORTING INFORMATION

Materials S2

Methods: Formulation, Transfection and Biophysical Studies S2

Vesicle Preparation S2

Formulation of Lipid:DNA Complexes S3

Dynamic Light Scattering and Zeta Potential S3

Gel Retardation Experiments S3

Picogreen Experiments S4

Transmission Electron Microscopy S4

Small Angle Neutron Scattering S4

Cell Culture S5

Transfection Experiments S5

Figures S7

Figure 1 SI S7

Figure 2 SI S8

Figure 3 SI S9

Figure 4 SI S10

Figure 5 SI S11

Figure 6 SI S12

Figure 7 SI S13

References S14

General Materials

N-[1-(2,3-dioleyloxy)propyl]-*N,N,N*-trimethyl-ammonium chloride (DOTMA) was supplied by TCI Europe N. V. (Zwijndrecht, Belgium) and *N*-[1-(2,3-Dioleoyloxy)propyl]-*N*,*N*,*N*-trimethylammonium chloride Salt (DOTAP) and dioleoyl L-α-phosphatidylethanolamine (DOPE) by Avanti Polar Lipids (Alabaster, Alabama, USA). Lipofectamine and Lipofectamine 2000 (L2K) were purchased from Invitrogen Life Technologies, UK. The synthesis of the lipids has been described in Mohammadi et al.[1] Calf thymus DNA (ctDNA) was purchased from Sigma (Dorset, UK) while the gWIZ-luciferase plasmid (pDNA) was obtained from Aldveron (Freiburg, USA). The luciferase assay kit was from Promega (Madison, Wisconsin, UK), and the BCA protein assay kit from Thermo scientific (Massachusetts, USA). Opti-MEM was purchased from Invitrogen Life Technologies (Massachusetts, UK). Fetal bovine serum (FBS), all other cell culture media and reagents and chemicals required for buffers, the gel retardation, release and protection assay were supplied by Sigma (Poole, Dorset, UK). All materials were of the highest grade available and were used as received. Rat neuroblastoma B104 cells were a gift from the Institute of Child Health, University College London. D_2O (>99.9% deuteration) was purchased from Sigma-Aldrich (Poole, Dorset, UK). Water was either deionised water that was double distilled in a well-seasoned still where the purity was regularly checked by surface tensiometry (a surface tension of 72.8 ± 1 mN m^{-1} at 20 ± 2 °C was deemed acceptable) and spectroscopically, or purified with a Millipore Milli-Q system to a resistivity of 18 MΩ cm. All water was filtered through 0.22 μM cellulose acetate filters (Millipore) prior to use.

Methods: Formulation, Transfection and Biophysical Studies

Vesicle Preparation

Cationic vesicles (1:1 molar ratio of cationic lipid and DOPE) were prepared using the thin film method. In brief 1 mg of cationic lipid and an equimolar quantity of DOPE were dissolved in chloroform and the solvent evaporated overnight *in vacuo* to produce a thin film of lipid. The film was subsequently hydrated with 1 mL of filtered water (or in the case of the small angle neutron scattering studies/circular dichroism/UV-Vis measurements, 1 mL D_2O) and the sample vortex mixed to produce a crude suspension of vesicles which were placed on ice and sonicated for 10 min (Lucas-Dawes probe sonicator (model: 7535A) fitted with a tapered microtip operating at 50% of maximum output). When sonicating vesicles dispersed in D_2O, the preparations were covered with Parafilm® to reduce the likelihood of D to H exchange. The sonicated vesicle suspension was centrifuged

(Beckman Microfuge, UK) at 13,000 rpm (~16,000 g) for 10 min to remove any titanium particles that had been shed from the probe.

Formulation of Lipid:DNA Complexes

Lipoplexes (or LDs) were formulated by adding equal volumes of DNA solution, (either ctDNA or pDNA) to the cationic vesicle suspension and gently mixing by hand to produce complexes with charge ratios in the range 1:1 - 3:1 cationic lipid:DNA (~ equivalent to 3:1 - 8:1 cationic lipid:DNA weight ratios). The LDs were left to stand for 15 min at room temperature before use. The final concentration of lipid and DNA in the LD depended on the experiment being performed.

Dynamic Light Scattering and Zeta Potential

The apparent hydrodynamic size and zeta potential of vesicle suspensions (containing 0.01 mg mL^{-1} of cationic lipid) and LDs (final DNA concentration of 1-2 µg of DNA per mL) were measured using a Zetasizer Nano-ZS series (Malvern Instruments Ltd, UK) at 25 ± 0.1 °C. At least 3, but usually 6 repeat measurements were performed on each sample, with each composition being samples on at least 2 but typically 3 occasions. When the apparent hydrodynamic size and zeta potential of vesicles and LDs prepared in D_2O were measured, the values of the refractive index and viscosity for D_2O at 25 °C were used in the analysis in place of the corresponding values of H_2O. Note that no difference in the apparent hydrodynamic size or zeta potential of the vesicles or LD complexes was observed when prepared in D_2O. A few samples were also prepared in pH 5 and pH 7.4 buffer. Furthermore, no differences were observed in the apparent hydrodynamic size or zeta potential of the complexes prepared using ct and pDNA. The effect of PBS and Opti-MEM on the LDs was investigated by mixing 50% vol of LDs in water with either double strength PBS or Opti-MEM.

Gel Retardation Experiments

The pDNA binding efficiency of LD complexes was determined using agarose gel electrophoresis (0.8% w/v agarose gel containing Gel Red in Tris-acetate-edetate (TAE) buffer at pH 7.4). LDs to be tested were formulated at L:D charge ratios of 1:1 to 4:1 and to a final pDNA concentration of 1 µg per 10 µL per well. 2 µL of gel loading buffer (prepared using 0.25% w/v bromphenol blue in a 40% w/v sucrose solution) was added to each sample before loading onto each well. The gel was run at 80 mV for one hour (Fisher Brand Model HU12 electrophoreses chamber, Loughborough, UK) after which the gel was visualized under UV light illumination using an AlphaImage EP Multi-Image Light Cabinet (Randpark Ridge, South Africa). Uncomplexed, free pDNA was used as a control. Each gel was repeated on more than one occasion to ensure reproducibility.

Picogreen Experiments

In an attempt to determine the extent of pDNA complexation, picogreen-binding to the LDs was measured. 50 µL of picogreen reagent (namely 1:150 v:v picogreen in 3 x Tris-EDTA (TE) buffer) was added to 100 µL of LD suspension (containing 0.2 µg of DNA) and incubated in 96-well black plates at room temperature for 5 min before fluorescence was determined. The fluorescence associated with the LDs was measured at an excitation wavelength of 485 nm and an emission wavelength of 520 nm with a gain of 1000 using a FLUOstar Omega fluorimeter (BMG LABTECH GmbH, Ortenberg, Germany) equipped with a plate reader. Any pDNA not incorporated in the LDs was quantified and expressed as relative fluorescence units (RFU) compared to the free (naked) pDNA control, which was denoted as 100% RFU in order to normalise the fluorescence signal. All experiments were performed in triplicate and the mean and SD calculated.

Transmission Electron Microscopy

Vesicle suspensions and LDs were negatively stained with 4 % w/v uranyl acetate by placing a drop of sample on a formvar 200 mesh copper grid for one minute followed by drying with filter paper. The grid was then placed on a drop of uranyl acetate for approximately five minutes and washed in 50% ethanol then in water and dried. The samples were visualised using an FEI Tecnai™ T12 transmission electron microscope (FEI Company, USA). For the electron microscopy studies LDs containing 0.05 mg mL^{-1} ctDNA and vesicles containing 1 mg mL^{-1} of cationic lipid were used.

Small Angle Neutron Scattering

Small angle neutron scattering (SANS) measurements were performed at the LOQ beam line at the ISIS pulsed neutron source (STFC Rutherford-Appleton Laboratory, Didcot, UK). Freshly prepared vesicles (1.0 mg mL^{-1}) and LD complexes (final ctDNA concentration of 0.05 mg mL^{-1}) were placed in clean, disc-shaped fused silica (Hellma, Southend on Sea, Essex, UK) cells of 2 mm path length and their SANS measured at 25 ± 0.1 °C. The SANS intensity, I(Q), of a sample, measured as a function of the scattering vector, Q = (4p/λ) sin(q/2), was normalised to the appropriate sample transmission. D_2O backgrounds were subtracted after normalisation of the solvent scattering to the solvent transmission. All fitting procedures included flat background corrections to account for any mismatch in the incoherent and inelastic scattering between the sample and the D_2O solvent. The levels of the fitted backgrounds were checked to ensure that they were of a physically reasonable magnitude.

Due to the size and polydispersity of the vesicles, the SANS data for the vesicles were analysed using the mixed 'sheet and stack model' in which the vesicles were assumed to be a mixture of infinite

planar sheets (taken to be the bilayer of any unilamellar vesicles present in the preparation) and one-dimensional stacks (taken to be the bilayer and repeat distance for any multilamellar vesicles present).[1] In all cases the model was constrained to yield the same mean thickness of the bilayer (L) for both the unilamellar and multilamellar vesicles. The SANS data was modelled by the least square refinement of up to 6 parameters, namely bilayer (L), Lorentz correction factor ($R\sigma$), the number of bilayers in a stack (M), their mean separation (D) and the absolute scale factors for the unilamellar and multilamellar vesicles. If no multilamellar vesicles were present only 3 parameters were refined. In the present study fitted value of $R\sigma$ (related to the curvature and extent of rigidity for the sheets) was in the range of 250 ± 20 unless otherwise stated. In all cases the polydispersity of the bilayer thickness ($\sigma(L)/L$) was fixed as 0.0001 for all the systems studied and (σ(D)/D]), the width of the Gaussian distribution in the plane, was fixed at 0.01. For all models, the least-squares refinements were performed using the model-fitting routines provided in the FISH software.[2]

Cell Culture

Rat neuroblastoma B104 cells were cultured in Dulbecco's Modified Eagle's medium (DMEM), supplemented with 10% v/v foetal bovine serum (FBS), 1% v/v L-glutamine 200mM, 1% v/v MEM-non-essential amino acids (NEAA), 0.2 mg/mL sodium pyruvate and 1% v/v penicillin/streptomycin solution at 37 °C, 5% CO_2 and 90% relative humidity. The cells were fed every other day and passaged every three to four days at around 70-80 % confluency, using 0.25% trypsin-EDTA solution.

Transfection Experiments

B104 cells were plated on 24-well plates at 8 x 10^4 cells per well and incubated at 37 °C, 5% CO_2 and 90% relative humidity for 24 hours. On the day of the experiment the DMEM growth medium was removed from each well, the cells rinsed once with 200 µL of PBS then replaced with 200 µL of Opti-MEM. Lipoplexes were prepared freshly by mixing equal volumes, usually 400 µL of the required lipid concentration with 400 µL of 0.01 mg mL^{-1} of pDNA in either Optimem or serum-containing media. The mixtures were left to complex for 15 minutes at room temperature, then 200 µL from the above mixtures were added to each of four wells containing 200 µL of Opti-MEM producing a final DNA concentration of 1 µg per 400 µL per well.

The plates were incubated at 37 °C for four hours after which the cells were washed once with PBS buffer and replaced with serum supplemented DMEM culture medium. The cells were left incubated for 48 hours, to allow for luciferase expression, which was measured using a luciferase assay kit according to manufacturer's protocols. Briefly, cells were lysed by the addition of 200 µL of a 1 x reporter lysis buffer (provided in the kit) for 1 hour at 37 °C before freezing at -70 °C for at least 30 minutes followed by thawing at room temperature to ensure more efficient cell lysis. 50 µL of the

lysate was then transferred to a white 96-well plate and the luciferase activity was measured for 10 seconds using an MLX Microtitre® Plate Luminometer (Dynex Technologies, USA) with an automatic feeding system delivering 100 µL of the reconstituted luciferase assay reagent into each well. The amount of protein in each transfection lysate was measured using a BCA protein assay kit according to manufacturer's instructions. Luciferase activity was expressed as Relative Light Units (RLU) per milligram of protein (RLU mg^{-1} protein).

Figures:

(a)

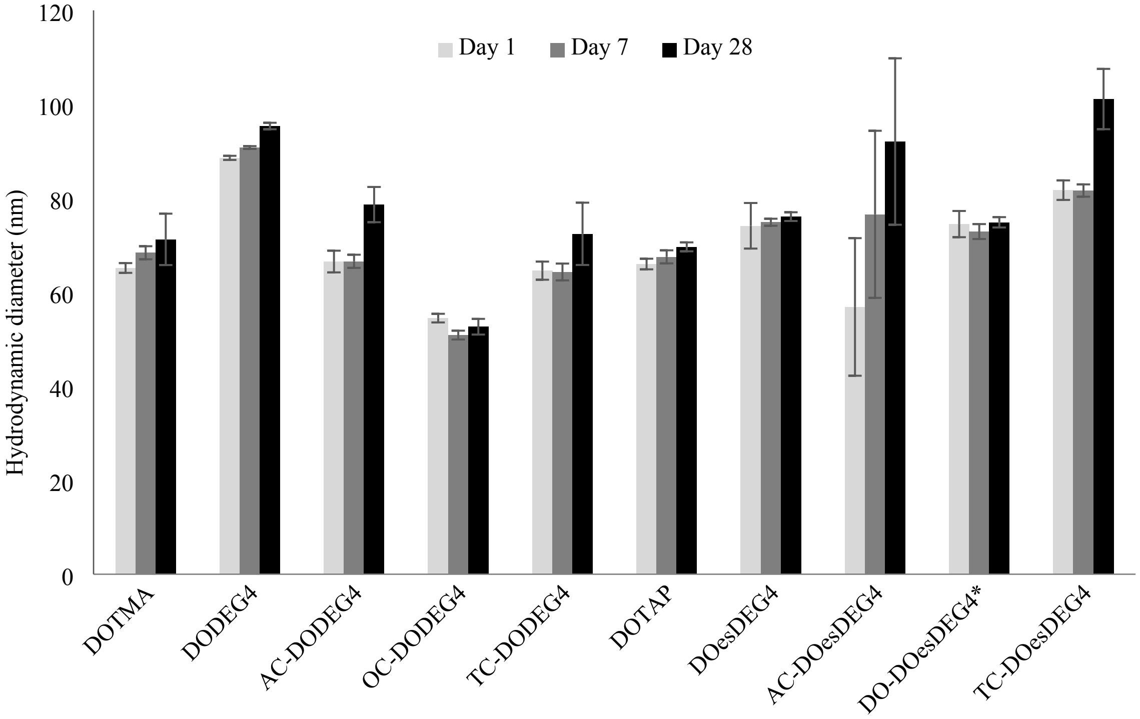

(b)

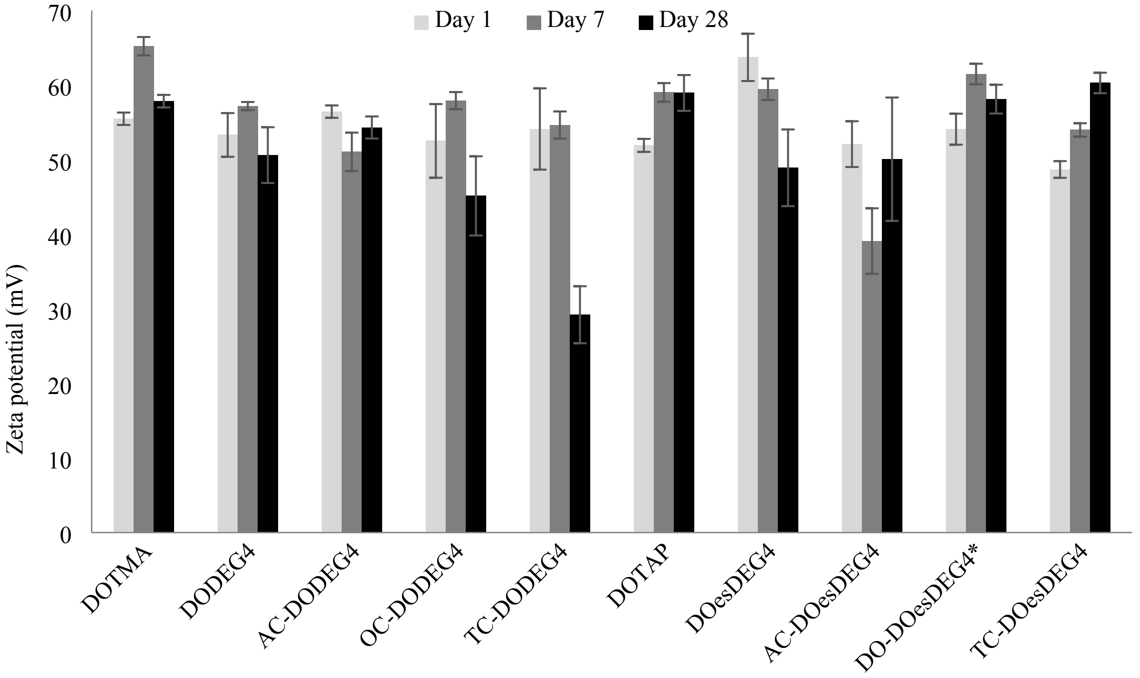

Figure 1 E**SI** The apparent hydrodynamic size (nm) (a) and zeta potential (mV) (b) of vesicles (prepared from various lipids in combination with DOPE or (*) DOPC) recorded at 298 ± 0.1 K. on day 1 (pale grey bars), day 7 (dark grey bars) and day 28 (black bars) after their preparation. Measurements were performed at a cationic lipid concentration of 0.01 mg/mL. All measurements were performed in triplicate and are shown as the mean value ± standard deviation.

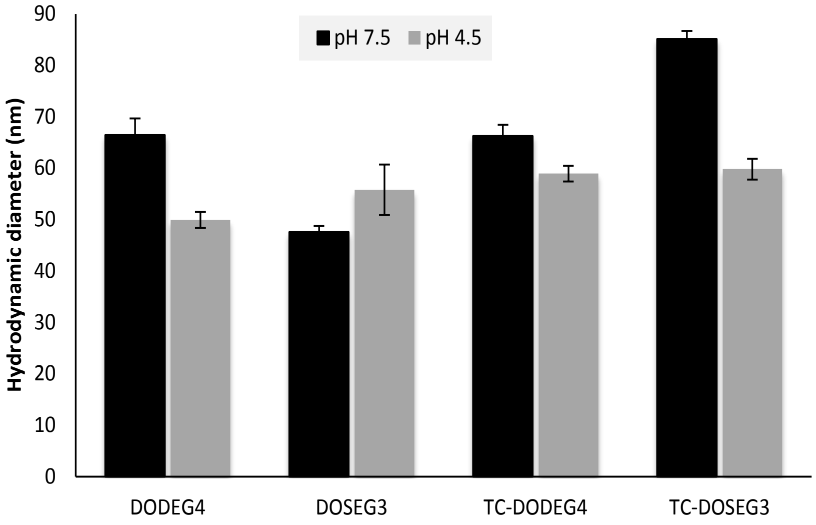

Figure 2 ESI: The apparent hydrodynamic size of vesicles prepared from the dichain (DC) cationic lipids, DODEG4 and DOSEG3 and their trichain (TC) prepared with an equimolar amount of DOPE in pH 7.5 and 4.5 at a cationic lipid concentration of 1 mg/mL and at a temperature of 298 ± 0.1 K. All measurements were performed in triplicate and are shown as the mean value ± standard deviation.

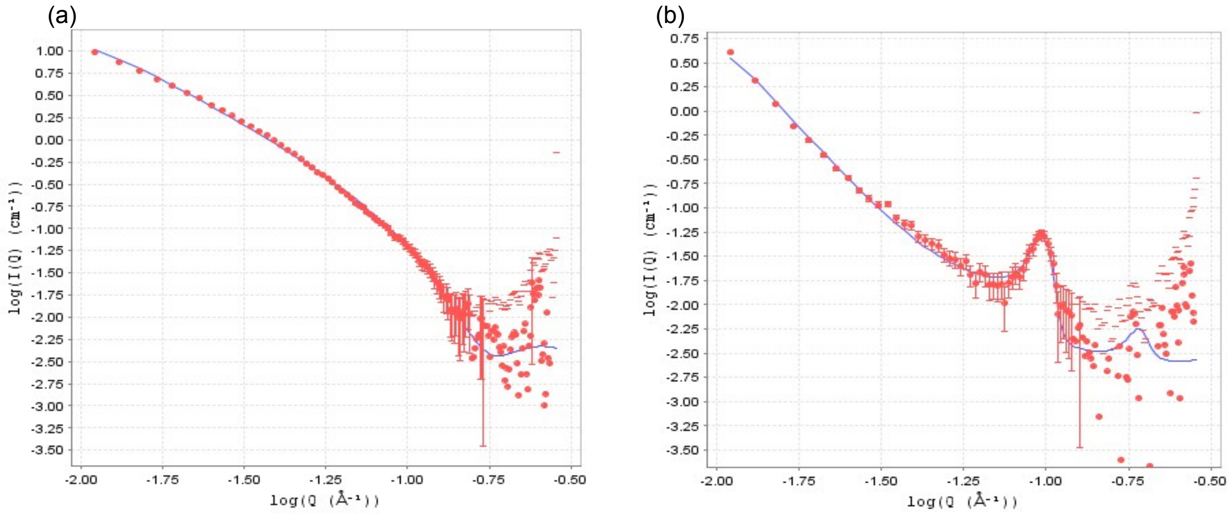

Figure 3 ESI: Small angle neutron scattering data (dots) recorded at 298 K and the best fit to the data (solid line) obtained assuming the sheet model for freshly prepared vesicles prepared from DOPE and 1:1 molar ratio of cationic lipid, Me-DOesDEG3 at a cationic lipid concentration of 1 mg/mL (a) and the mixed sheet and stack model for corresponding lipoplexes (LDs) prepared from the same vesicvles and ctDNA at a L:D charge ratio of 2:1.

(a)

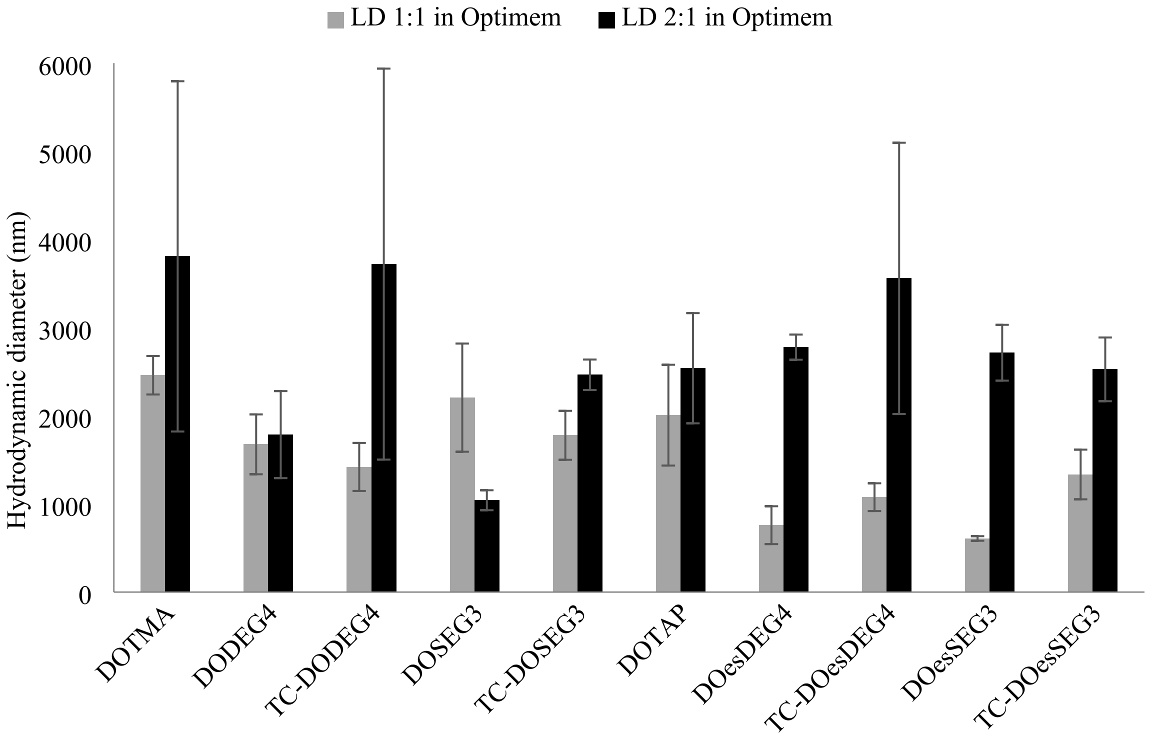

(b)

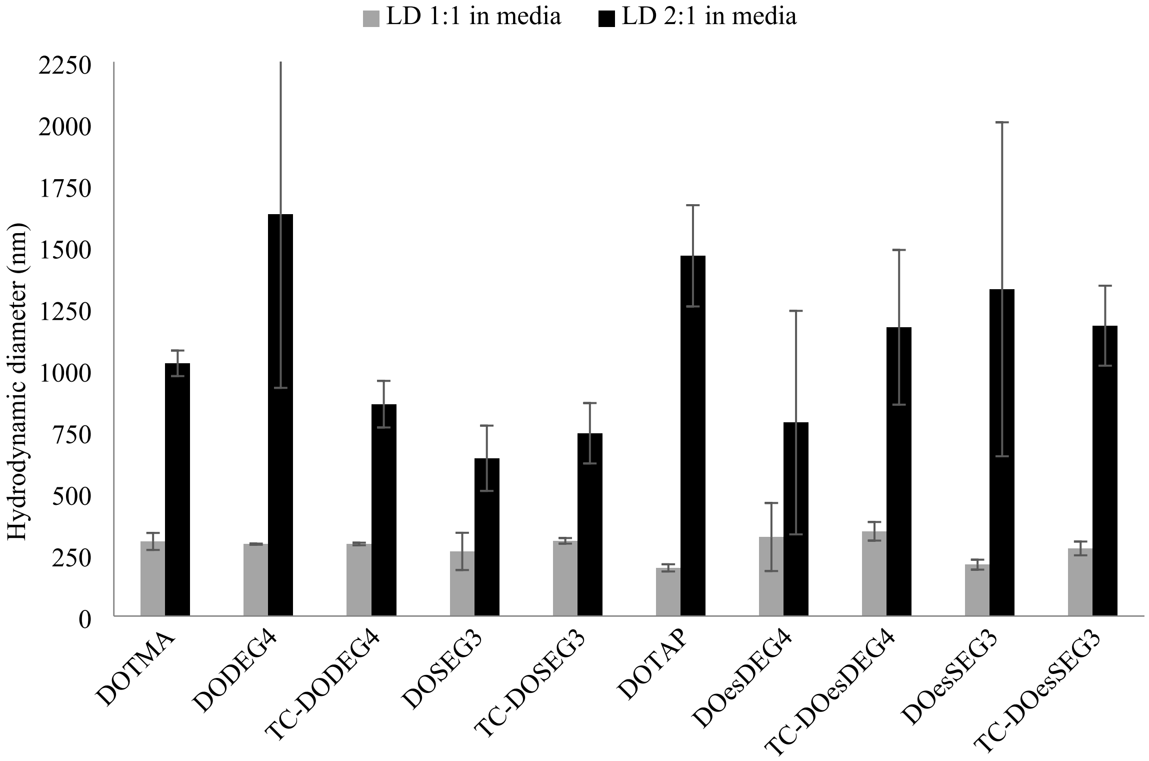

Figure 4 ESI: The apparent hydrodynamic size (nm) of lipoplexes (LDs), prepared from vesicles of various lipids in combination with DOPE using ctDNA at L:D charge ratios of either 1:1 or 2:1 charge ratios when added to (a) Opti-MEM or (b) FBS-containing media at 298 ± 0.1 K. All measurements were performed in triplicate and are shown as the mean value ± standard deviation.

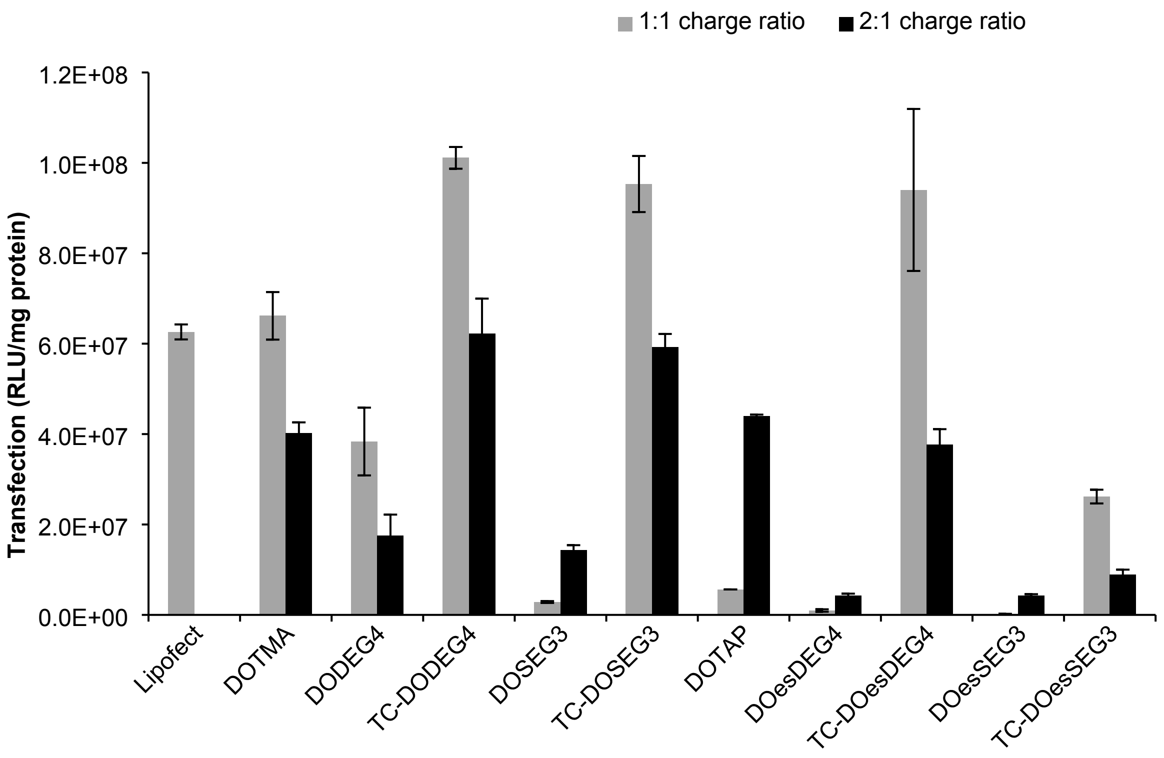

Figure 5 ESI. In vitro transfection efficiency (expressed as RLU/mg protein) of LD complexes prepared in Opti-MEM at 1:1 (grey bars) and 2:1 (black bars) lipid:gWiz plasmid DNA charge ration in N2A cells. Cationic lipid used to prepare the LDs shown on the abscissa. All the lipids were formulated at a 1:1 ratio with DOPE. Data is the mean of three measurements ± standard deviation.

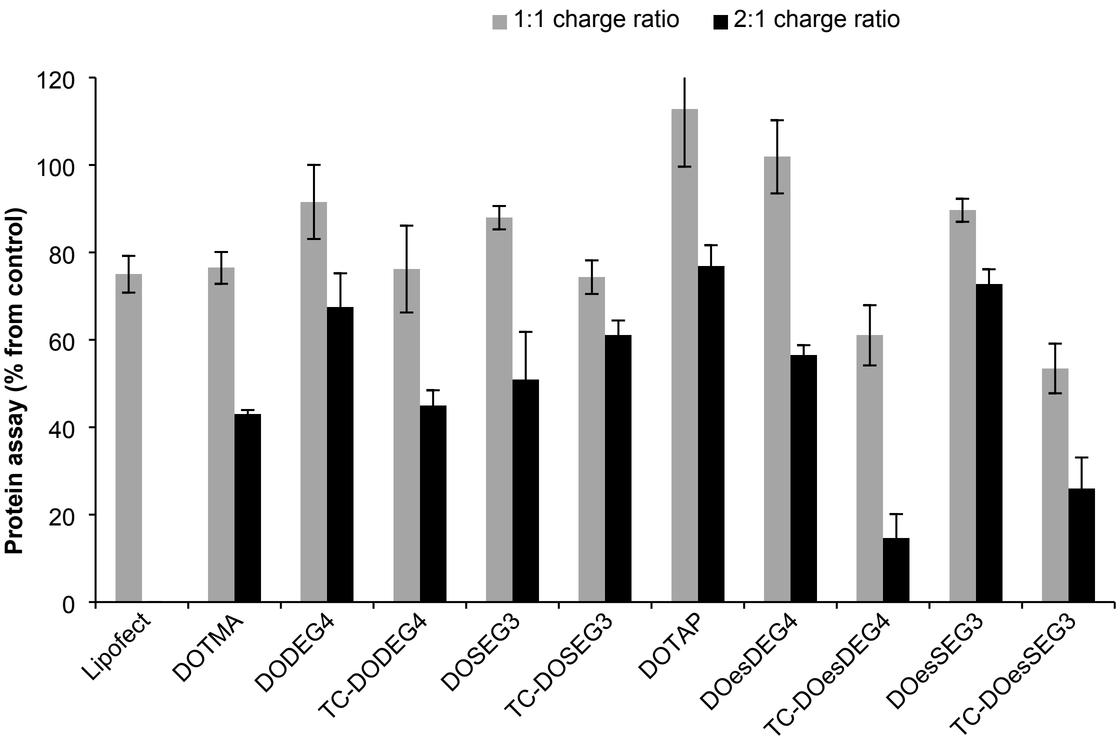

Figure 6 ESI. Protein assay of LD complexes prepared in Opti-MEM at 1:1 (grey bars) and 2:1 (black bars) lipid:gWiz plasmid DNA charge ratio in N2A cells. Cationic lipid used to prepare the LDs shown on the abscissa. All the lipids were formulated at a 1:1 ratio with DOPE. Data is the mean of three measurements ± standard deviation.

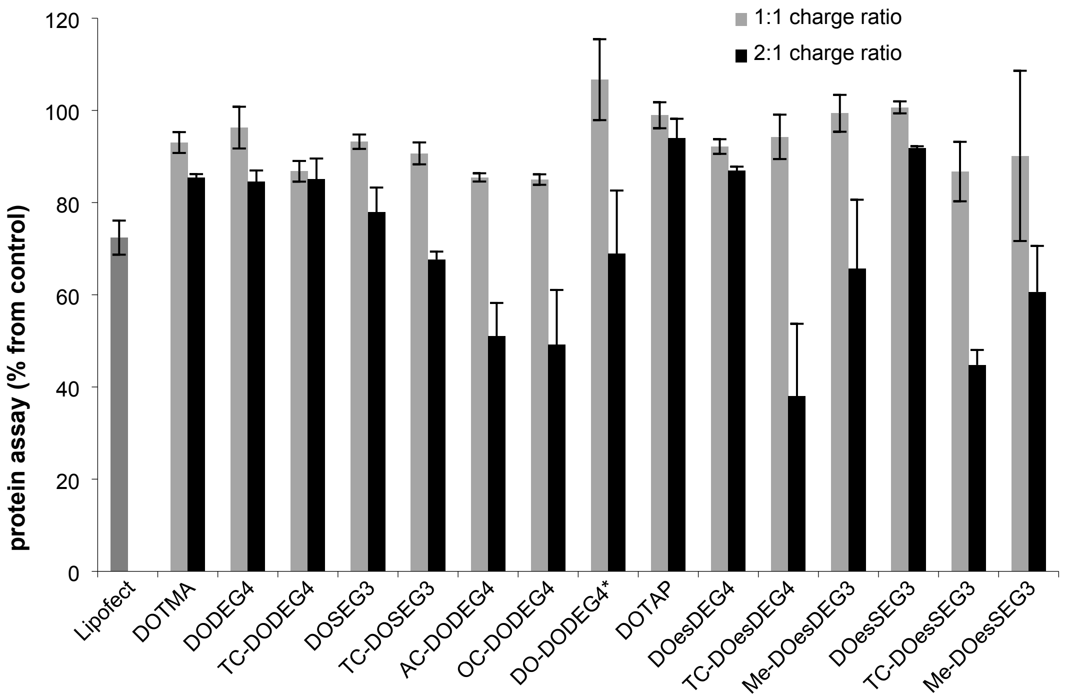

Figure 7 ESI: Protein assay (expressed as % from untreated control) of LD complexes prepared in Opti-MEM at 1:1 (grey bars) and 2:1 (black bars) lipid:gWiz plasmid DNA charge ratio in B104 cells. Cationic lipid used to prepare the LDs shown on the abscissa. All the lipids were formulated at a 1:1 ratio with DOPE or (*) DOPC. Data is the mean of three measurements ± standard deviation.

References

(1) Atefah Mohammadi, Laila Kudsiova, M. Firouz M. Mustapa, Frederick Campbell, Katharina Welser, Danielle Vlaho, Harriet Storey, Aristides D. Tagalakis, Stephen L. Hart, David J. Barlow, Alethea B. Tabor, M. Jayne Lawrence and Helen C. Hailes (2017) Trichain lipids: discovery, synthesis, characterization and application in gene delivery systems.

(2) R. K. Heenan, 1989. The “Fish” reference manual. Data fitting program for small-angle 716 neutron scattering. RAL report 89-129 (Revised 2000), Rutherford Appleton Laboratory, UK.

Note: reference 1 will need to match ref 8 of the main paper
